# Suitability of Different Analytical Derivations of Electrically Induced Stress States in Planar and Cylindrical Dielectric Elastomer Actuators

**DOI:** 10.3390/ma15041321

**Published:** 2022-02-10

**Authors:** Sascha Pfeil, Gerald Gerlach

**Affiliations:** Institute of Solid-State Electronics, Faculty of Electrical and Computer Engineering, Technische Universität Dresden, 01069 Dresden, Germany; gerald.gerlach@tu-dresden.de

**Keywords:** dielectric elastomer actuators, soft robotics, electrically induced stress states, analytical stress derivations, electro-mechanical coupling, cylindrical DEA, tube actuators

## Abstract

Dielectric elastomers (DE) belong to a very performant and efficient class of functional materials for actuators, while being compliant, low-weight and silent, they offer high energy efficiencies and large deformations under an applied electric field. In this work, a comparison of different approaches to derive expressions for the electrically induced stress states in dielectric materials is given. In particular, the focus is on three different ways to analytically describe stress states in planar actuator setups and to show how they are connected to each other regarding their resulting deformations. This is the basis to evaluate the suitability of these approaches for cylindrical actuator geometries together with exemplary calculations for concrete use cases. As an outcome, conclusions on the suitability of the different approaches for certain actuator setups are drawn. In particular cylindrical actuator geometries are taken into account and a recommendation on which approach is useful to describe a certain actuator effect is given.

## 1. Introduction

Electroactive polymers (EAP) and the related dielectric elastomer actuators (DEAs) are offering great potential for soft robotic applications and unconventional actuator setups. Current developments are generating an impressive innovative momentum with ongoing improvements in the field of soft robotics and drive concepts.

For methodical descriptions of actuator effects in general it is necessary to formulate a suitable analytical description of their actuation impact. In the case of DEAs this is the electrically induced stress state in a dielectric material, leading to a deformation in a certain spatial direction.

For the most simple case of a planar actuator setup, consisting of a dielectric film, sandwiched between two compliant electrodes, the directly resulting stress state from the attraction of the electrodes towards each other is in thickness direction of the dielectric film. From an engineering point of view that stress component will not sufficiently describe a desired actuation effect in the planar spatial directions and needs certain additional considerations. When it comes to planar elongations of the dielectric film itself, a description in exactly the planar directions is needed, also to compare it to results of force measurements from tensile tests.

A possible method to derive such spatial stress states in dielectric materials is to make use of the so-called Maxwell stress tensor. As stated by Goulbourne [1], the Maxwell stress tensor renders the observable electrical force between charged bodies and lacks of electrostriction in a way that a coupled change of dielectric constants to mechanical deformations is not considered. To include the Maxwell stress into continuum descriptions, Toupin [2] introduced an approach to include local elastic stress states and electrically induced stress states to render the total Cauchy-stress.

Focusing on that approach, the here presented work aims to specify the electrically induced stress states for different actuator geometries to specifically close the gap between electrically induced stress states in deformable materials and continuum descriptions for chosen actuator geometries.

Especially for cylindrical DEA setups, different approaches have been proposed. In general, cylindrical setups have been developed as rolled actuators and tube actuators, while rolled actuators consist of planar dielectrics that are rolled up to form a cylindrical geometry as presented in various publications [3,4,5], tube actuators are built as a seamless dielectric film that is fabricated in the cylindrical geometry. Here, we exclusively focus on such tube actuators and therefore refer to them when speaking of cylindrical actuators in the following.

Though that special actuator geometry is an emerging topic, there are various examples to be found in the literature [6,7,8]. Most of the published work [9,10,11] describes the electro-mechanical behavior of tube actuators by following an approach to utilize the stored electrostatic energy in a system under certain boundary conditions, especially volume incompressibility. Together with mechanical constitutional descriptions, the internal elastic stress state is taken into account to formulate a combined electro-mechanical description.

Here, alongside with some established approaches, a method to describe the electrically induced stress state in dielectric materials, depending only on electric field variables is proposed. Together with methods of continuum mechanics, the derived stress can be used to model electro-mechanical behavior of deformable dielectric materials. In order to give a clear insight into purely electrically induced effects as they occur in classical dielectric materials, no consideration of anisotropic material behavior and cross-influences of thermal or strain-dependent processes is given here.

In the following, we point out how to describe electrically induced stress states in DEAs. In the first part the focus is on the well-described planar setups and how different derived stress states are linked to each other as a review. The following part is about the investigation of these methods for cylindrical actuator geometries. Since some different conditions apply for the planar case, some limitations occur, which we investigate and give advice on which approach can be useful for a certain case. In particular, the following considerations will be pointed out.

- The electrostatic pressure acting on planar DEA electrodes follows certain boundary conditions that are not directly applicable for more complex geometries.
- In planar actuator geometries, the material can be modeled to be uniformly deformed under an applied electric field in thickness direction. The resulting deformation takes place in all free planar directions.
- The underlying stress state in the dielectric material can be described based on the approaches considered in this work. For planar actuator geometries these stress states can be described as dependent on only one geometrical variable. The resulting stress state is valid for the whole geometry.
- Cylindrical actuator geometries show field properties, that not only make the approaches based on the descriptions for planar DEAs more complex. Some of the approaches even can not be used to analytically describe the behavior of the macroscopic geometry.

## 2. Model Approaches

In this section, an overview on three different methods to derive expressions for electrically induced stress states in dielectric materials is given. The underlying assumptions for both considered actuator geometries are the following.

- The electrodes needed to apply an electric field are fully compliant and perfectly conductive. Therefore they are neglected in concern of mechanical and electrical influences.
- The applied charges are assumed to be homogeneous distributed over the electrodes.
- The resulting electric field between the electrodes is assumed to consist only of components normal to the electrode area. No side effects at the edges of the electrodes are assumed to be existent.
- The electrodes are assumed to be connected perfectly to the dielectric material without any effects of an interface layer.
- The dielectric material is considered as incompressible, isotropic and hyperelastic.
- The separated charges on the electrodes are of the same value with inverted polarizations Q and −Q.
- Furthermore, we make use of the convention to shortly write ε for the mathematical expression $\varepsilon =\varepsilon_{r}\cdot \varepsilon_{0}$ with the vacuum permittivity $\varepsilon_{0}$ and the relative dielectric permittivity of the material $\varepsilon_{r}$.

**Figure 1:**
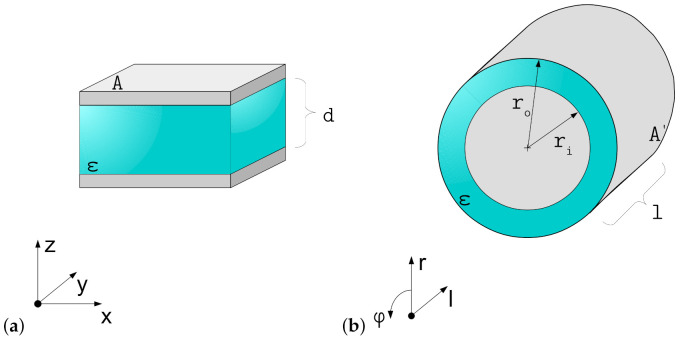
Considered actuator setups: (**a**) plate capacitor, representing a planar DEA and (**b**) coaxial capacitor, representing a cylindrical DEA.

For the further descriptions both planar and coaxial actuator setups are considered as shown in Figure 1. The models presented here are developed for planar actuator structures and transferred to cylindrical structures.

### 2.1. Stored Energy Approach

The concept of utilizing the change of the stored electrostatic energy $w_{\mathrm{el}}$ in a capacitor was introduced by Pelrine and Kornbluh in 1998 [12] for planar DEAs. They described the electrical induced pressure in thickness direction by formulating the total differential of the electrostatic energy depending on the electrode area *A* and the film thickness *d*. In general $w_{\mathrm{el}}$ can be described as dependent on the charge Q and the applied voltage *V* according to Sahdev [13] as

(1) $$w_{\mathrm{el}}=\frac{1}{2}\cdot Q\cdot V.$$

That formulation is valid for both considered geometries and can be regarded as an equivalent description for the work that was necessary to separate the charges between the electrodes. Together with constitutive descriptions for *V* in the system, a tailored equation for each actuator geometry can be found.

Since the energy function can be considered as a description of the performed work on the dielectric, that function can be used to find an expression for the force acting on an electrode. In contrast to Pelrine’s approach of using the total differential, we suggest to make use of the boundary condition of incompressibility and to formulate an expression that only depends on one geometrical variable. In the planar case this could be *d*, leading to a description of the force impact in thickness direction as

(2) $$f_{z}=\frac{dw_{\mathrm{el}}}{dd}.$$

The stress acting in z-direction is then found as

(3) $$\sigma_{z}=\frac{f_{z}}{A}.$$

### 2.2. Energy Balance Approach

Another way to describe an electrically induced stress state in a DEA was presented in 2007 by Wissler et al. [14]. The presented approach suggests to use the energy balance in a system of a plate capacitor and letting it change for the volume-depending variables by deriving the single energy contributions for the corresponding variables. To formulate the energy balance, the input energy $w_{\mathrm{in}}$ as electrical energy from the source, the electrostatic energy $w_{\mathrm{el}}$ as described before and the mechanical energy $w_{\mathrm{mech}}$ due to the movement of the flexible setup are taken into account. The advantage of this approach is that a formulation for $w_{\mathrm{mech}}$ can be found out of the other two formulations, which are described in a matter of electrical values. As for the stored energy approach it is again useful to formulate the single contributions as only depending on one geometrical variable, in this case exemplary on *d*. The energy balance can be formulated as

(4) $$\frac{dw_{\mathrm{in}}}{dd}=\frac{dw_{\mathrm{el}}}{dd}+\frac{dw_{\mathrm{mech}}}{dd},$$

resulting in the mechanical energy derivation, which is also an expression for the force impact on the electrodes

(5) $$\frac{dw_{\mathrm{mech}}}{dd}=\frac{dw_{\mathrm{in}}}{dd}-\frac{dw_{\mathrm{el}}}{dd}=f_{z}.$$

The electric input energy follows in general the description of

(6) $$w_{\mathrm{in}}=V\cdot I$$

with the electric current I that can be described as a change of charge in terms of a change in thickness *d* due to volume incompressibility as

(7) $$I=\frac{dQ}{dd}.$$

Both the expressions for Q and *V* depend on the considered actuator geometry and have to be adapted, respectively, under the boundary condition of volume incompressibility to be dependent on only one variable. The contribution from the electrostatic energy $w_{\mathrm{el}}$ follows the same principles as described before.

### 2.3. Maxwell Stress Tensor Approach

According to textbooks about electromagnetism and basics of electrical engineering [15,16,17], the stress state on a unit volume of dielectric material subjected to an electric field can be expressed, being only dependent on field values. These values render the so called Maxwell stress tensor, that describes the resulting stress components in all spatial directions inside the dielectric material. Assuming the absence of magnetic fields and describing only a static case, the Maxwell stress tensor is defined as:

(8) $$\vec{T}=\epsilon (\vec{E}\cdot \vec{E^{T}}-\frac{1}{2}\cdot I\cdot {\left|E\right|}^{2}).$$

Together with the field descriptions for the considered geometrical cases, the Maxwell stress tensor will deliver concrete values for the stress components in the spatial directions. Since this is a differential description, it is necessary to transform it into a description for the macroscopic geometry by calculating a force component that acts on the whole geometrical area in the desired direction. It can be calculated by integrating, respectively, over the area element $d\vec{A}$ for that direction.

(9) $$\vec{F}=\oint \vec{T}\cdot d\vec{A}$$

Afterwards a macroscopic stress component in the spatial direction *i* can be formulated by dividing the force component ${\vec{F}}_{i}$ over the area $A_{i}$.

(10) $$\sigma_{i}=\frac{{\vec{F}}_{i}}{A_{i}}.$$

### 2.4. Resulting Deformations and Equivalent Stress States

In general, the different approaches are valid as long as they can be properly described according to the geometrical conditions and as long as the boundary conditions are not violated. That means that for a fairly simple system of a planar DEA all three approaches are valid and they just vary regarding the component-wise composition of their results. Suo pointed out in 2010 [18] that under the assumed boundary conditions of liquid-like behavior and the geometrical conditions of a planar DEA, an introduced hydrostatic pressure p can connect the derived stress states from the different approaches. The hydrostatic pressure acts universal on a system in every spatial direction with the same value and translates the uniaxial stress state $\vec{u}$ to the triaxial stress state $\vec{t}$ by superposition and vice versa.

(11) $$\vec{u}=\vec{p}+\vec{t}$$

The superposition approach can be used to show that it is possible to regard the different stress states as equivalent. The fact that a hydrostatic pressure could be chosen arbitrarily to result in the same deformation state makes that approach less intuitive. Any other vector for *p* would be still correct as long as it consists of same values for its components but it would deliver another expression out of the superposition. A more intuitive approach is to underpin the outcome of equivalent stress states by taking the stress-induced deformation of the dielectric material into account. To describe the deformation, Hooke’s law for linear elasticity in isotropic materials is suitable. It describes the component-wise spatial strains $\epsilon_{i}$ resulting out of a given stress state $\sigma_{i}$ and the material’s Young’s modulus Y. Note, that a load on a material in one direction always causes stress in only that direction but strains in all free directions. Under the assumption of a Poisson ratio of ν = 0.5, Hooke’s law can be expressed as:

(12) $$\left(\begin{array}{c} \epsilon_{x}\\ \epsilon_{y}\\ \epsilon_{z} \end{array}\right)=\frac{1}{Y}\left(\begin{array}{ccc} 1 & -\nu & -\nu \\ -\nu & 1 & -\nu \\ -\nu & -\nu & 1 \end{array}\right)\left(\begin{array}{c} \sigma_{x}\\ \sigma_{y}\\ \sigma_{z} \end{array}\right).$$

Using these component-wise expressions, the different results from the presented approaches can be inserted for $\sigma_{i}$ to check if different stress states lead to same deformation states and hence can be regarded as equivalent. Table 1 gives an overview of the different assumptions, boundary conditions and characteristics of the three different approaches.

## 3. Planar Actuator Geometries

**Figure 2:**
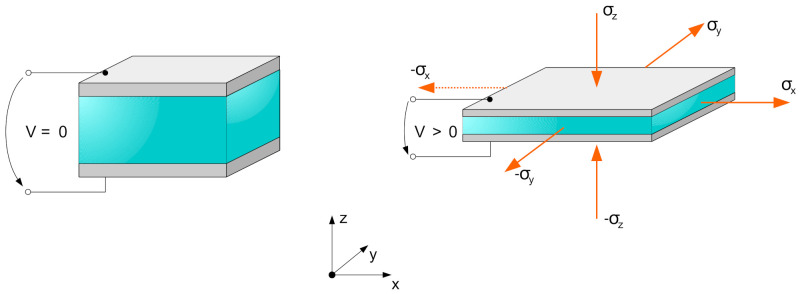
Principle of a planar DEA with spatial stress states and resulting deformation for an activated DEA.

In this section, concrete examples for stress states in planar geometries are derived using the methods presented before. The aim is to point out which approach is useful for a certain desired result. First, the case of a planar DEA is considered. The principle of such a DEA is shown in Figure 2, together with the resulting spatial stress states.

Under the mentioned assumptions from Section 2, the actuator geometry can be modeled as an incompressible rectangular cuboid. The dielectric material inside the DEA consists of an elastomer such as silicone that shows a liquid-like behavior in a way that it can be regarded as fully elastic and incompressible. The resulting deformation out of the electrical activation can be described using a deformation gradient tensor F that consists of the spatial stretch ratios $\lambda_{i}$ as following.

(13) $$F=\left(\begin{array}{ccc} \lambda_{x} & 0 & 0\\ 0 & \lambda_{y} & 0\\ 0 & 0 & \lambda_{z} \end{array}\right)$$

**Figure 3:**
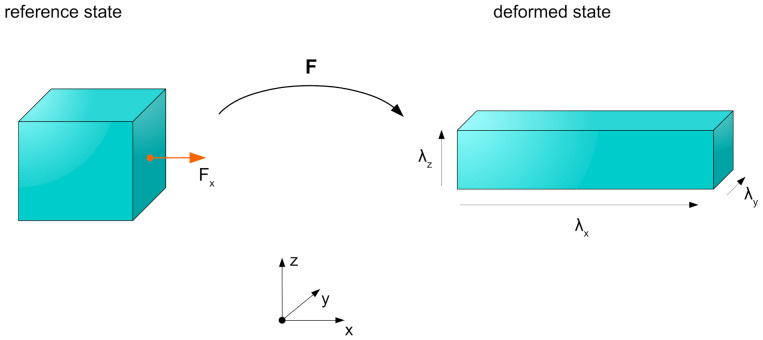
Deformation states for the incompressible material, connected via the deformation gradient tensor.

Figure 3 depicts the considered deformation of the dielectric material and the conncetion via F between the unactivated state as reference configuration and the activated state as deformed configuration.

The deformation of an incompressible material follows the condition that the product of the principle stretch ratios remains constant with a value of one. The Jacobian determinant J is expressed as:

(14) $$J=\lambda_{1}\cdot \lambda_{2}\cdot \lambda_{3}\equiv 1.$$

Using that connection, a simplification for the electrostatic energy function can be found to express it as only dependent on *d*. The volume vol of the dielectric material can be described as

(15) vol=A·d=constant.

Using that connection, the variable *A* in the description of the electrostatic energy can be substituted with

(16) $$A=\frac{\mathrm{vol}}{d}.$$

For the planar case some constitutional electrical equations apply, that specify the following considerations. The constitutional equations for a plate capacitor are introduced here as

(17) Q=C·V,

(18) $$C=\epsilon \cdot \frac{A}{d},$$

and

(19) $$V=\frac{Q\cdot d}{\epsilon \cdot A},$$

with the capacitance C of the capacitor. Equation (18) inserted into Equation (17) gives

(20) $$Q=\epsilon \cdot \frac{A}{d}\cdot V.$$

with the description for *V* from Equation (19) together with *A* from Equation (16) the expression for $w_{\mathrm{el}}$ from Equation (1) gives

(21) $$w_{\mathrm{el}}=\frac{1}{2}\cdot \frac{Q^{2}}{\epsilon \cdot \mathrm{vol}}\cdot d^{2}.$$

In this way all degrees of freedom for the deformation are considered and the function is described as only depending on one variable.

Under these prerequisites, the electrostatic energy approach can be carried out. By deriving $w_{\mathrm{el}}$ for *d*, an expression for the electrically generated force on the electrodes acting in thickness direction is found as

(22) $$f_{z}=\frac{dw_{\mathrm{el}}}{dd}=\frac{Q^{2}}{\epsilon \cdot \mathrm{vol}}\cdot d.$$

Resubstituting the volume expression from Equation (15) gives

(23) $$f_{z}=\frac{Q^{2}}{\epsilon \cdot A}.$$

Inserting the constitutional Equation (20) for Q specifies the acting force on the electrodes as

(24) $$f_{z}=\epsilon \cdot A\cdot {\left(\frac{V}{d}\right)}^{2}$$

and the stress acting in z-direction is calculated as

(25) $$\sigma_{z}=\frac{f_{z}}{A}=\epsilon \cdot {\left(\frac{V}{d}\right)}^{2}.$$

Next, the energy balance approach is carried out for the case of a planar DEA. The electrical input energy contribution can be formulated under the previously described substitutions as

(26) $$w_{\mathrm{in}}=V\cdot \frac{dQ}{dd}$$

and together with *V* from Equation (19) and *A* from Equation (16) $w_{\mathrm{in}}$ gives

(27) $$w_{\mathrm{in}}=\frac{Q^{2}\cdot d^{2}}{\epsilon \cdot \mathrm{vol}}\frac{d}{dd}=\frac{dw_{\mathrm{in}}}{dd}.$$

As in the previous approach, the electrostatic energy contribution is found as

(28) $$w_{\mathrm{el}}=\frac{1}{2}\cdot \frac{Q^{2}}{\epsilon \cdot \mathrm{vol}}\cdot d^{2}.$$

Using these energy contributions, the energy balance can be formulated according to Equation (4) as

(29) $$\frac{Q^{2}\cdot d^{2}}{\epsilon \cdot \mathrm{vol}}\frac{d}{dd}=\frac{1}{2}\cdot \frac{Q^{2}}{\epsilon \cdot \mathrm{vol}}\cdot d^{2}\frac{d}{dd}+\frac{dw_{\mathrm{mech}}}{dd}.$$

Carrying out the derivations and reformulating according Equation (5) gives an expression for the mechanical energy derivation, which is equivalent to a force as

(30) $$f_{z}=\frac{dw_{\mathrm{mech}}}{dd}=\epsilon \cdot A\cdot {\left(\frac{V}{d}\right)}^{2}.$$

**Figure 4:**
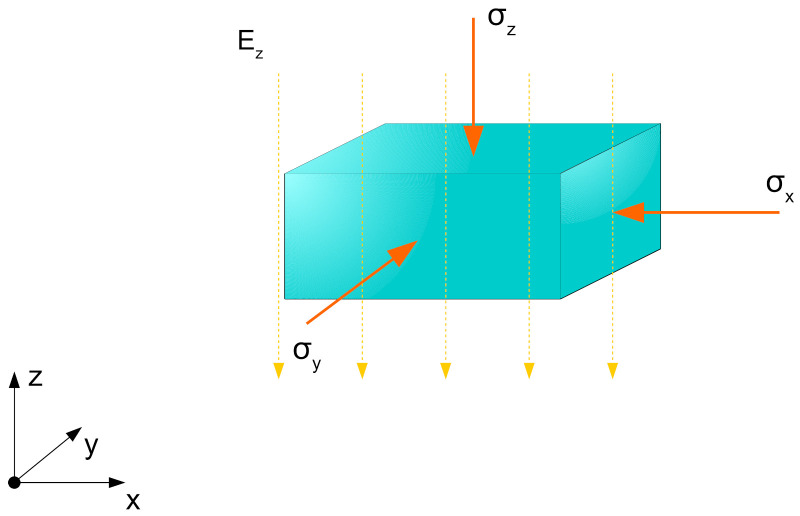
Stress state according to the Maxwell stress tensor approach for a unit volume element in an electric field distributed exclusively in z-direction.

The corresponding stress is again calculated as

(31) $$\sigma_{z}=\frac{f_{z}}{A}=\epsilon \cdot {\left(\frac{V}{d}\right)}^{2}$$

which is the same as for the electrostatic energy approach. To carry out the Maxwell stress tensor approach, first a description of the electric field is necessary. The applied electric field in z-direction for the planar actuator setup follows the illustration in Figure 4.

The electric field is assumed to be homogeneously distributed and can be written as

(32) $$\vec{E}=\left(\begin{array}{c} 0\\ 0\\ E_{z} \end{array}\right).$$

with that field vector the principal components of the Maxwell stress tensor give

(33) $${\vec{T}}_{\mathrm{xx}}=\epsilon \cdot \left(E_{x}^{2}-\frac{1}{2}\cdot E_{z}^{2}\right)=-\frac{1}{2}\cdot \epsilon \cdot E^{2},$$

(34) $${\vec{T}}_{\mathrm{yy}}={\vec{T}}_{\mathrm{xx}}=-\frac{1}{2}\cdot \epsilon \cdot E^{2},$$

and

(35) $${\vec{T}}_{\mathrm{zz}}=\epsilon \cdot \left(E_{z}^{2}-\frac{1}{2}\cdot E_{z}^{2}\right)=\frac{1}{2}\cdot \epsilon \cdot E^{2}.$$

Due to the homogeneous field distribution in the cuboid geometry, the determination of a force component that acts on the geometry’s area is not necessary and in this case the components can be interpreted as macroscopic stresses

(36) $${\vec{T}}_{\mathrm{ij}}=\sigma_{\mathrm{ij}}.$$

The remarkable outcome of that description is that here, an expression for the stress state in thickness direction of exact half the value as derived with the other approaches occurs. Table 2 gives an overview of the derived stress components from the different approaches for planar actuator geometries.

Using Hooke’s law as described before, it is possible to check if these stress states result in same deformation states. For both the energy approaches the deformation strains give

(37) $$\left(\begin{array}{c} \epsilon_{x}\\ \epsilon_{y}\\ \epsilon_{z} \end{array}\right)=\left(\begin{array}{c} \frac{1}{Y}\cdot \left(-\frac{1}{2}\cdot \epsilon \cdot {\left(\frac{V}{d}\right)}^{2}\right)\\ \frac{1}{Y}\cdot \left(-\frac{1}{2}\cdot \epsilon \cdot {\left(\frac{V}{d}\right)}^{2}\right)\\ \frac{1}{Y}\cdot \left(\epsilon \cdot {\left(\frac{V}{d}\right)}^{2}\right) \end{array}\right).$$

For the triaxial stress state from the Maxwell stress tensor approach the deformation strains give

(38) $$\left(\begin{array}{c} \epsilon_{x}\\ \epsilon_{y}\\ \epsilon_{z} \end{array}\right)=\left(\begin{array}{c} \frac{1}{Y}\cdot \left(-\frac{1}{2}\cdot \epsilon \cdot {\left(\frac{V}{d}\right)}^{2}\right)\\ \frac{1}{Y}\cdot \left(-\frac{1}{2}\cdot \epsilon \cdot {\left(\frac{V}{d}\right)}^{2}\right)\\ \frac{1}{Y}\cdot \left(\epsilon \cdot {\left(\frac{V}{d}\right)}^{2}\right) \end{array}\right).$$

which is exactly the same as for the uniaxial stress state. That underpins the statement, that the derived stress states for the different approaches are equivalent and valid besides the fact that they consist of different values.

## 4. Cylindrical Actuator Geometries

To give an overview of the presented approaches for more complex geometries, a cylindrical geometry representing a fiber actuator is considered and the stress states according to the presented methods are calculated. We make use of the following additional assumptions:

- The inner radius $r_{i}$ is fixed.
- The electrical activation leads to a reduction of the outer radius $r_{o}$ and a thinning of the dielectric layer.
- The resulting deformation exclusively takes place in length direction and radial direction.

Furthermore it is suitable to make use of the cylindrical geometry and describe the equations in cylindrical coordinates with

- $x_{1}$→ radial direction r
- $x_{2}$→ angular direction ϕ
- $x_{3}$→ length direction l

**Figure 5:**
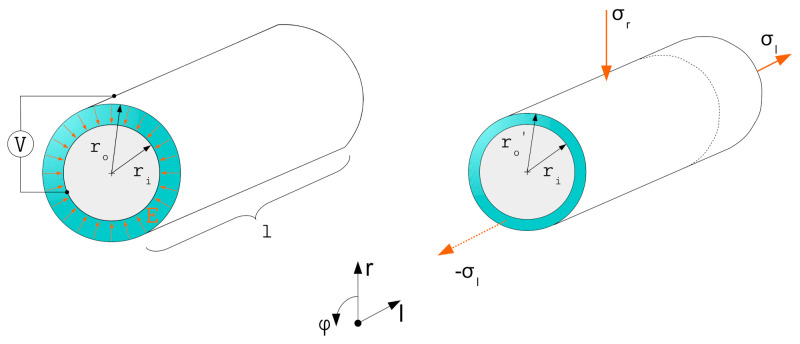
Concept of a cylindrical actuator geometry with field distribution inside the dielectric material and resulting deformations under the assumed conditions.

Figure 5 shows the concept of the considered geometry.

The cylindrical actuator geometry needs some adapted constitutional equations that have to be taken into account. The incompressible volume of the dielectric material follows the conditions:

(39) $$\mathrm{vol}=\pi \cdot (r_{o}^{2}-r_{i}^{2})\cdot l$$

and

(40) $$l=\frac{\mathrm{vol}}{\pi \cdot (r_{o}^{2}-r_{i}^{2})}.$$

The electric field is described as

(41) $$E=\frac{Q}{\varepsilon \cdot A}=\frac{Q}{\varepsilon \cdot 2\pi \cdot r\cdot l}$$

which can be used to find a description for the charge Q in terms of the voltage V, using the electric potential $\Phi_{o}$ of the outer electrode and $\Phi_{i}$ of the inner electrode

(42) $$\Phi_{o}=\Phi_{i}-\int_{r_{i}}^{r_{o}}E\left(r\right)dr,$$

(43) $$\Phi_{o}=\Phi_{i}-\frac{Q}{\varepsilon \cdot 2\pi \cdot l}\cdot \ln\left(\frac{r_{o}}{r_{i}}\right).$$

Out of the general description for an electrical voltage the equation can be adapted to

(44) $$V=\Phi_{o}-\Phi_{i}=-\frac{Q}{\varepsilon \cdot 2\pi \cdot l}\cdot \ln\left(\frac{r_{o}}{r_{i}}\right),$$

leading to

(45) $$V=\frac{Q}{\varepsilon \cdot 2\pi \cdot l}\cdot \ln\left(\frac{r_{i}}{r_{o}}\right)$$

and

(46) $$Q=V\cdot \frac{\varepsilon \cdot 2\pi \cdot l}{\ln(\frac{r_{i}}{r_{o}})}.$$

Here it is important to notice that in contrast to the planar case, the electric field is distributed non-homogeneously over the radius, resulting in a radius-dependent voltage between the electrodes. To execute the electrostatic energy approach, first it is necessary to formulate the electrostatic energy function

(47) $$w_{\mathrm{el}}=\frac{1}{2}\cdot Q\cdot V=\frac{1}{2}\cdot \frac{Q^{2}}{\varepsilon \cdot 2\pi \cdot l}\cdot \ln\left(\frac{r_{i}}{r_{o}}\right).$$

Using the constitutive Equation (40) that results in

(48) $$w_{\mathrm{el}}=\frac{1}{2}\cdot \frac{Q^{2}\cdot \pi \cdot \left(r_{o}^{2}-r_{i}^{2}\right)}{\varepsilon \cdot 2\pi \cdot \mathrm{vol}}\cdot \ln\left(\frac{r_{o}}{r_{i}}\right)=\frac{1}{4}\cdot \frac{Q^{2}\cdot \left(r_{o}^{2}-r_{i}^{2}\right)}{\varepsilon \cdot \mathrm{vol}}\cdot \ln\left(\frac{r_{i}}{r_{o}}\right).$$

The force impact on the surface area due to the applied field is again described as the derivative of the electrostatic energy for the variable *r*. Since *r* does not occur in the equation, deriving means shifting the variable $r_{o}$ and therefore also changing the scaling value of $\ln(\frac{r_{i}}{r_{o}}$ for the equation. The important difference to the planar geometry is, that for every shifted $r_{o}$, another solution occurs since the electric field is not homogeneously distributed along the radius and the result is only valid on the area at the position $r_{o}$. Nevertheless, we carry out the derivation as follows for $r_{o}$ to give an exemplary result for the first step of a possible iterative numerical solution as

(49) $$f_{r}=\frac{dw_{\mathrm{el}}}{dr_{o}}=\frac{Q^{2}}{4\varepsilon \cdot \mathrm{vol}}\cdot \frac{d}{dr_{o}}\left[\left(r_{o}^{2}-r_{i}^{2}\right)\cdot \ln\left(\frac{r_{i}}{r_{o}}\right)\right].$$

Applying the product rule delivers

(50) $$f_{r}=\frac{Q^{2}}{4\varepsilon \cdot \mathrm{vol}}\cdot \left[2\cdot r_{o}\cdot \ln\left(\frac{r_{i}}{r_{o}}\right)-\left(r_{o}^{2}-r_{i}^{2}\right)\cdot \frac{1}{r_{o}}\right].$$

The resulting stress state in radial direction, acting on the outer electrode at $r_{o}$ is given by

(51) $$\sigma_{r}=\frac{f_{r}}{A}=\frac{f_{r}}{2\pi \cdot l\cdot r_{o}}.$$

Resubstitution of vol from Equation (39) and inserting the constitutional Equation (46) for Q leads to

(52) $$\sigma_{r}=\varepsilon \cdot \frac{V^{2}\cdot \left[2\cdot r_{o}\cdot \ln\left(\frac{r_{i}}{r_{o}}\right)-\left(r_{o}^{2}-r_{i}^{2}\right)\cdot \frac{1}{r_{o}}\right]}{2\cdot \ln^{2}\left(\frac{r_{i}}{r_{o}}\right)\cdot r_{o}\cdot \left(r_{o}^{2}-r_{i}^{2}\right)}.$$

As already mentioned, that stress state is only valid at the exact position of the outer electrode at $r_{o}$. To find a solution for resulting deformations, the derived stress state would need to be evaluated numerically by calculating the result iterative until the solution converges. An analytical solution for the whole system is not given by this approach. Since the energy balance approach uses the same description of the electrostatic energy $w_{\mathrm{el}}$, it will also be only valid at the exact position of the outer electrode at $r_{o}$ and not for the whole geometry. Therefore we leave it out here. Using the general definition of the Maxwell stress tensor, it becomes possible to describe the differential stress state in the dielectric material under the given assumptions. The acting stress components in the spatial directions can be formulated as:

(53) $${\vec{T}}_{r}=\varepsilon \cdot \left(E_{r}^{2}-\frac{1}{2}\cdot E_{r}^{2}\right)=\frac{1}{2}\cdot \varepsilon \cdot E_{r}^{2},$$

(54) $${\vec{T}}_{\phi }=-\frac{1}{2}\cdot \varepsilon \cdot E_{r}^{2},$$

and

(55) $${\vec{T}}_{l}=-\frac{1}{2}\cdot \varepsilon \cdot E_{r}^{2}.$$

Using the field description from Equation (41) and the constitutive Equation (46) for the charge Q, the radial component of the Maxwell stress tensor is given by

(56) $${\vec{T}}_{r}=\frac{1}{2}\cdot \varepsilon \frac{V^{2}}{\ln^{2}\left(\frac{r_{o}}{r_{i}}\right)\cdot r^{2}}.$$

The force acting on a radial area element $d{\vec{A}}_{r}$ results out of

(57) $$f_{r}=\underset{A}{\;\int \int \;}{\vec{T}}_{r}\cdot d{\vec{A}}_{r}$$

with the differential radial area element

(58) $$d{\vec{A}}_{r}=r\cdot d\phi \cdot dl\cdot {\vec{e}}_{r}.$$

The force component in radial direction results in

(59) $$f_{r}=\frac{1}{2}\cdot \varepsilon \cdot \frac{V^{2}}{\ln^{2}\left(\frac{r_{o}}{r_{i}}\right)\cdot r}\int_{0}^{l}\int_{0}^{2\pi }d\phi \cdot dl=\varepsilon \cdot l\cdot \pi \cdot \frac{V^{2}}{\ln^{2}\left(\frac{r_{o}}{r_{i}}\right)\cdot r}.$$

Using that description for the integral force component it is possible to calculate an averaged mean stress in radial direction $\sigma_{r,\mathrm{avg}}$ as

(60) $$\sigma_{r,\mathrm{avg}}=\frac{f_{r}}{A_{r}}$$

with

(61) $$A_{r}=2\cdot \pi \cdot r\cdot l.$$

$\sigma_{r,\mathrm{avg}}$ results in

(62) $$\sigma_{r,\mathrm{avg}}=\frac{1}{2}\cdot \varepsilon \cdot \frac{V^{2}}{\ln^{2}\left(\frac{r_{o}}{r_{i}}\right)\cdot r^{2}},$$

which is the same as the direct output from the Maxwell stress tensor. The stress component in angular direction is calculated similarly as:

(63) $${\vec{T}}_{\phi }=-\frac{1}{2}\cdot \varepsilon \frac{V^{2}}{\ln^{2}\left(\frac{r_{o}}{r_{i}}\right)\cdot r^{2}},$$

using the corresponding area element in angular direction $d{\vec{A}}_{\phi }$

(64) $$d{\vec{A}}_{\phi }=dr\cdot dl\cdot {\vec{e}}_{\phi }.$$

The resulting force acting in angular direction can be described as:

(65) $$f_{\phi }=-\frac{1}{2}\cdot \varepsilon \frac{V^{2}}{\ln^{2}\left(\frac{r_{o}}{r_{i}}\right)}\int_{0}^{l}\int_{r_{i}}^{r_{o}}\frac{1}{r^{2}}dr\cdot dl=\frac{1}{2}\cdot \varepsilon \cdot \frac{V^{2}\cdot l}{\ln^{2}\left(\frac{r_{o}}{r_{i}}\right)\cdot \left(r_{o}-r_{i}\right)}.$$

The averaged mean stress in angular direction $\sigma_{\phi ,\mathrm{avg}}$ is calculated as

(66) $$\sigma_{\phi ,\mathrm{avg}}=\frac{f_{\phi }}{A_{\phi }}$$

with

(67) $$A_{\phi }=\left(r_{o}-r_{i}\right)\cdot l.$$

$\sigma_{\phi ,\mathrm{avg}}$ results in

(68) $$\sigma_{\phi ,\mathrm{avg}}=\frac{1}{2}\cdot \varepsilon \cdot \frac{V^{2}}{\ln^{2}\left(\frac{r_{o}}{r_{i}}\right)\cdot {\left(r_{o}-r_{i}\right)}^{2}},$$

which is not the same as the direct output from the Maxwell stress tensor.

The stress component in length direction is the same as in angular direction

(69) $${\vec{T}}_{l}=-\frac{1}{2}\cdot \varepsilon \frac{V^{2}}{\ln^{2}\left(\frac{r_{o}}{r_{i}}\right)\cdot r^{2}}.$$

Using the corresponding area element in length direction $d{\vec{A}}_{l}$

(70) $$d{\vec{A}}_{l}=r\cdot dr\cdot d\phi \cdot {\vec{e}}_{l},$$

the force acting in length direction can be described as:

(71) $$f_{l}=-\frac{1}{2}\cdot \varepsilon \frac{V^{2}}{\ln^{2}\left(\frac{r_{o}}{r_{i}}\right)}\int_{0}^{2\pi }\int_{r_{i}}^{r_{o}}\frac{1}{r}dr\cdot d\phi =-\varepsilon \cdot \frac{V^{2}\cdot \pi }{\ln(\frac{r_{o}}{r_{i}})}.$$

The averaged mean stress in length direction $\sigma_{l,\mathrm{avg}}$ is calculated as

(72) $$\sigma_{l,\mathrm{avg}}=\frac{f_{l}}{A_{l}}$$

with

(73) $$A_{l}=\pi \cdot \left(r_{o}^{2}-r_{i}^{2}\right).$$

$\sigma_{l,\mathrm{avg}}$ results in

(74) $$\sigma_{l,\mathrm{avg}}=-\varepsilon \cdot \frac{V^{2}}{\ln\left(\frac{r_{o}}{r_{i}}\right)\cdot \left(r_{o}^{2}-r_{i}^{2}\right)},$$

which is also not the same as the direct output from the Maxwell stress tensor. An overview of the results is given in Table 3.

## 5. Validity Comparison for Cylindrical Geometries

Based on the well-established descriptions of electrically induced stress states for planar actuator geometries, it would be comprehensible to simply use the descriptions for planar actuator setups inserted with a film thickness resulting from the difference between the inner and outer radius to describe a stress state. Despite the fact that this approach, as already described, does not provide an exact solution for cylindrical actuator geometries, the approach will be compared with the correct analytical solution at this point to give an estimate of the deviation between the approaches as a function of geometry ratios.

To evaluate the outcome and the deviation between the approaches, the averaged Maxwell stress component in length-direction from Equation (74) and the stress component in length-direction based on the electrostatic energy from Equation (33) are considered. Together with given values for $\varepsilon_{0}=8.854187\cdot 10^{-12}\frac{A\cdot s}{V\cdot m}$, $\varepsilon_{r}=2.7$, an applied voltage of 5000 V and an assumed thickness of the dielectric of 50 μm, which represent typical values for silicone-based dielectrics, a function plot for $\sigma_{l,\mathrm{avg}}$ can be obtained.

**Figure 6:**
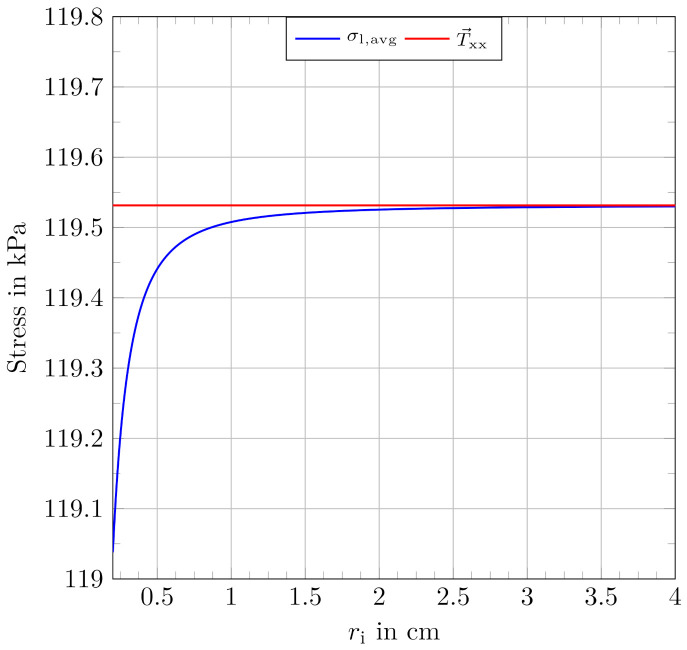
Typical function plot for $\sigma_{l,\mathrm{avg}}$ with given values as described and an assumed film thickness of 50 μm together with the constant result from the electrostatic approach, using the same input values.

With the given film thickness, ${\vec{T}}_{\mathrm{xx}}$ is calculated as a constant value for comparison. Figure 6 shows the results from the mentioned approaches for the given values as a typical function plot for $r_{i}$ in a range from 200 μm to 4 cm.

The combined plot suggests that a certain ratio of $r_{i}$ and $r_{o}$ exists, at which the approaches can be regarded as nearly equivalent and the outcome is in a comparable range. We suggest to introduce a band, which defines the correct values to be within ranges of 95%, 99% and 99.9% of the (incorrect) result from the planar approach. Within these boundaries, the results from the different approaches can be regarded as equivalent. Outside of these boundaries, a use of the correct approach, as presented in this paper, is strongly indicated. The boundary values $lim_{i}$ to determine the criteria as a concrete radius ratio result from the planar approach as:

(75) $$lim_{1}=0.95\cdot {\vec{T}}_{\mathrm{xx}}$$

(76) $$lim_{2}=0.99\cdot {\vec{T}}_{\mathrm{xx}}$$

and

(77) $$lim_{3}=0.999\cdot {\vec{T}}_{\mathrm{xx}}.$$

By reformulating the expressions for $\sigma_{l,\mathrm{avg}}$ and ${\vec{T}}_{\mathrm{xx}}$ to be dependent only on $r_{i}$ and the film thickness *d*, a numerical solution for the radius ratio *R* with

(78) $$R=\frac{r_{i}}{r_{o}},$$

that represents the corresponding thresholds can be found. The stress expressions are therefore reformulated to

(79) $$\sigma_{l,\mathrm{avg}}=\varepsilon \cdot \frac{V^{2}}{\ln\left(\frac{r_{i}+d}{r_{i}}\right)\cdot \left({\left(r_{i}+d\right)}^{2}-r_{i}^{2}\right)},$$

and

(80) $${\vec{T}}_{\mathrm{xx}}=\frac{1}{2}\cdot \epsilon \cdot \frac{V^{2}}{d^{2}}$$

and the balance equation to be solved results in

(81) $$\sigma_{l,\mathrm{avg}}=lim_{i}.$$

Iteratively setting values for *d* from 10 μm to 1 mm, which is a typical range for dielectric films, enables Equation (81) to be solved for $r_{i}$. Using each of the derived values for $r_{i}$, a radius ratio R according to Equation (78) can be calculated. Performing the described numerical calculations, the ratios *R* result in the values given in Table 4.

Using that outcome, corresponding value combinations of film thicknesses and inner radii can be calculated to mark the limits for the results from the correct approach from Equation (74) to be within the defined deviation of the approach from Equation (33). By assigning discrete values for *d*, the corresponding inner radii can be calculated as

(82) $$r_{i}=\frac{d\cdot R}{1-R}.$$

**Figure 7:**
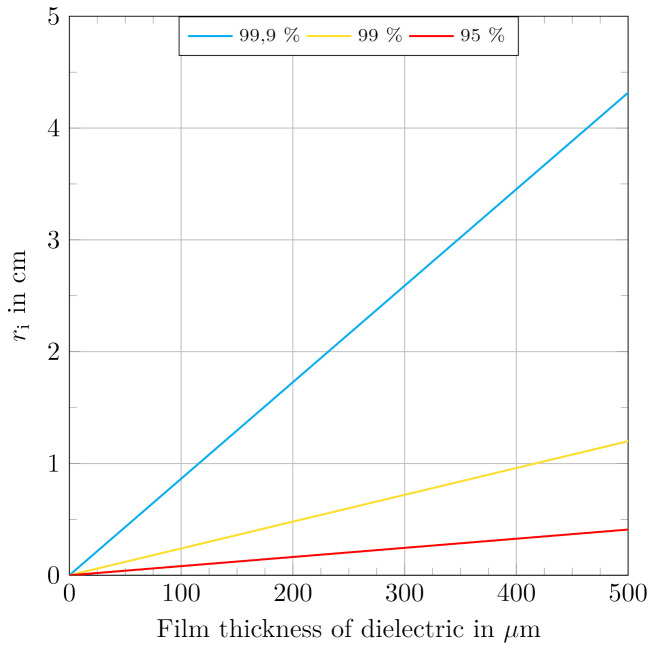
Limits for dielectric film thicknesses and corresponding inner radii of cylindrical actuator geometries to indicate the use of the correct formulation resulting from the Maxwell stress tensor to describe a stress state. Curves represent boundaries for the correct results to be within defined maximum deviations from the planar approach. Areas under the curves strongly indicate the use of the correct approach for $\sigma_{l,\mathrm{avg}}$.

Figure 7 shows the curve plots for the different defined limits applied to film thicknesses in a range from zero to 500 μm and the corresponding inner radii from zero to 5 cm.

With regard to that outcome, especially for cylindrical actuators with small diameters it becomes important to use the adapted equation for $\sigma_{l,\mathrm{avg}}$. For example, an actuator, utilizing a 100 μm dielectric could only be calculated with both expressions equivalently if its inner diameter is above 860 μm to result in values that differ by less than 0.1% from each other.

From an inverse engineering approach, an inner radius of the structure of 1 cm allows for a minimum film thickness of 115.8 μm to be calculated with both approaches to result in values that differ by less than 0.1% from each other. Film thicknesses below that strongly indicate the use of the correct equation for $\sigma_{l,\mathrm{avg}}$.

Considering as an example a concrete actuator setup, we assume a tube-actuator of an inner radius of 100 μm and a film thickness of 50 μm, fabricated of a typical silicone dielectric with relative permittivity $\varepsilon_{r}$ of 2.7 it is operated at a driving voltage of 5000 V. Utilizing Equation (33) from the planar approach, a resulting stress state of 119.53 kPa in length direction is induced under the described assumptions. Using the correct calculation from Equation (74) leads to a result of 117.92 kPa, acting in length direction. This means that the deviation from the incorrect approach amounts to 1.36%. Therefore, for that considered actuator, the use of the approach given in Equation (74) is strongly recommended. Assuming the same setup with an increased inner radius of 1 mm while keeping all other parameters at the same values results in 119.50 kPa for the correct approach and 119.53 kPa for the adapted approach from planar geometries, which represents a neglectable 0.025% deviation.

## 6. Comparison of Approaches and Conclusions

The presented approaches and considerations give a clear insight on how stress states can be calculated for planar and for cylindrical actuator geometries. In general the spatial oriented stress states can be derived using the presented methods for the exact desired use case. For planar geometries we have shown that making use of geometrical boundary conditions and material behavior, some comparatively simple approaches are suitable to derive a stress state in the dielectric material. For cylindrical actuator setups we draw the following conclusions.

- For cylindrical geometries the approach of using the differential of the stored electrostatic energy to describe a stress state for the whole geometry is not possible as in the planar case. The differential describes only the stress acting on the outer surface of the actuator setup at $r_{o}$, not for the whole geometry.
- The electric field distribution inside a cylindrical actuator is non-homogeneous. A deformation due to a previously derived stress state out of the electrostatic energy approach leads to changed geometrical conditions that also change the value of the electric field strength, making the stress description invalid. The approach becomes only applicable for a numerical problem solving.
- The cylindrical setup has one more degree of freedom in its electrical properties. Due to an assumed reduction of the outer radius $r_{o}$ the field strength inside the dielectric material is increased. To date, this is the same as in a planar setup. The applied voltage between the electrodes in contrast is also affected since it is derived from the electric field description which is again connected to the radius *r*.
- In contrast to the electrostatic energy approaches, the Maxwell stress tensor approach delivers a component-wise expression for the stress states in the dielectric material. For cylindrical geometries these results also depend on *r* but the integration over a differential area element gives a force value that describes the acting force analytically. Dividing that by the geometrical area of the setup gives an average stress state that is valid for the whole geometry and can be used for engineering approaches.
- Especially for scaled cylindrical actuator geometries with low diameters and film thicknesses, the use of the presented approaches based on the Maxwell stress tensor is strongly indicated. Especially for small actuator geometries, the resulting stress shows a highly non-linear behavior.

Out of the presented considerations and exemplary calculations we suggest to use the Maxwell stress tensor approach to describe electrically induced stress states in dielectric materials, especially for cylindrical actuator geometries. 

## Figures and Tables

**Table 1:** Overview of the assumptions, boundary conditions, applicability, advantages and disadvantages of the different approaches to derive electrically induced stress states in dielectric materials.

	Stored Energy Approach	Energy Balance Approach	Maxwell Stress Tensor Approach
Assumptions	Homogeneous field distribution Resulting stress component in field direction	Input energy results from a connected power supply Same as for stored energy approach	Electrical field properties can be described for the whole geometry Non-disturbed electrical field, oriented normal to the electrode area
Boundary conditions	Linear connection between electrostatic energy and geometric dimensions necessary Volume incompressibility	Mechanical energy contributions in the system can be formulated as resulting from only electrical energy contributions which are fully described and differentiable	Isotropic, homogeneous dielectric material Static case Absence of megnetic fields
Applicability	To describe a stress acting on the whole geometry in Planar actuator setups To describe surface stress state in cylindrical geometries	For electro-mechanical systems with direct connection to a power supply For energy contributions that can be formulated as dependent on the same geometrical variables	For homogeneous and inhomogeneous field distributions To derive a triaxial stress-state acting on a differential volume element
Advantages	Fast and intuitive approach based on geometrical conditions	Resulting mechanical energy differential, representing a performed work, can be described as exclusively dependent on electrical values	Delivers stress components in all spatial directions, directly applicable to describe actuator behavior in the desired direction
Disadvantages	Only valid for analytical descriptions in planar geometries Inhomogeneous field distributions lead to derived stress states that are not valid for the whole geometry	Only valid for analytical descriptions in planar geometries Inhomogeneous field distributions lead to derived stress states that are not valid for the whole geometry	Delivers a differential description, which for more complex geometries has to be integrated in a next step

**Table 2:** Resulting stress states out of the previously considered approaches for planar actuator geometries.

	Stored Energy Approach	Energy Balance Approach	Maxwell Stress Tensor Approach
$\sigma_{x}$	0	0	$-\frac{1}{2}\cdot \epsilon \cdot {\left(\frac{V}{d}\right)}^{2}$
$\sigma_{y}$	0	0	$-\frac{1}{2}\cdot \epsilon \cdot {\left(\frac{V}{d}\right)}^{2}$
$\sigma_{z}$	$\epsilon \cdot {\left(\frac{V}{d}\right)}^{2}$	$\epsilon \cdot {\left(\frac{V}{d}\right)}^{2}$	$\frac{1}{2}\cdot \epsilon \cdot {\left(\frac{V}{d}\right)}^{2}$

**Table 3:** Overview of the resulting stress states from the direct Maxwell stress tensor approach, the resulting force impacts and the averaged Maxwell stress components acting on the whole cylindrical geometry.

Component	Maxwell Stress	Maxwell Force	Averaged Maxwell Stress
radial component	$\frac{1}{2}\cdot \varepsilon \cdot \frac{V^{2}}{\ln^{2}\left(\frac{r_{o}}{r_{i}}\right)\cdot r^{2}}$	$\varepsilon \cdot l\cdot \pi \cdot \frac{V^{2}}{\ln^{2}\left(\frac{r_{o}}{r_{i}}\right)\cdot r}$	$\frac{1}{2}\cdot \varepsilon \cdot \frac{V^{2}}{\ln^{2}\left(\frac{r_{o}}{r_{i}}\right)\cdot r^{2}}$
angular component	$-\frac{1}{2}\cdot \varepsilon \cdot \frac{V^{2}}{\ln^{2}\left(\frac{r_{o}}{r_{i}}\right)\cdot r^{2}}$	$\frac{1}{2}\cdot \varepsilon \cdot \frac{V^{2}\cdot l}{\ln^{2}\left(\frac{r_{o}}{r_{i}}\right)\cdot \left(r_{o}-r_{i}\right)}$	$\frac{1}{2}\cdot \varepsilon \cdot \frac{V^{2}}{\ln^{2}\left(\frac{r_{o}}{r_{i}}\right)\cdot {\left(r_{o}-r_{i}\right)}^{2}}$
length component	$-\frac{1}{2}\cdot \varepsilon \cdot \frac{V^{2}}{\ln^{2}\left(\frac{r_{o}}{r_{i}}\right)\cdot r^{2}}$	$-\varepsilon \cdot \frac{V^{2}\cdot \pi }{\ln(\frac{r_{o}}{r_{i}})}$	$-\varepsilon \cdot \frac{V^{2}}{\ln\left(\frac{r_{o}}{r_{i}}\right)\cdot \left(r_{o}^{2}-r_{i}^{2}\right)}$

**Table 4:** Overview of the limiting radius ratios R for the different boundary definitions. The values result from numerical solutions for film thicknesses in a range between 10 μm and 1 mm.

Accuracy	95% Boundary	99% Boundary	99.9% Boundary
R	0.450	0.706	0.896

## Data Availability

Not applicable.

## Abbreviations

The following abbreviations are used in this manuscript:

DE	Dielectric elastomer
EAP	Electroactive polymer
DEA	Dielectric elastomer actuator
